# Stock-outs of antiretroviral drugs and coping strategies used to prevent changes in treatment regimens in Kinondoni District, Tanzania: a cross-sectional study

**DOI:** 10.1186/2052-3211-7-3

**Published:** 2014-04-22

**Authors:** Amani Thomas Mori, Joyce Owenya

**Affiliations:** 1 Department of Pharmaceutics, School of Pharmacy, Muhimbili University of Health and Allied Sciences, P.O. Box 65013, Dar es Salaam, Tanzania

**Keywords:** ART, Pharmaceutical systems, Resource-poor settings, Coping strategies, Stock-outs, Shelf-lives

## Abstract

**Objectives:**

Since 2004, the government of Tanzania has been rolling out antiretroviral treatment programs all over the country. However, the capacity of the health system to cope with the rapid scale-up of these programs is a major concern, and problems may result in drug stock-outs that force changes in treatment regimens. This study aims to explore stock-outs of antiretroviral drugs and further determine the coping strategies employed to prevent changes in treatment regimens in HIV/AIDS care and treatment clinics in Kinondoni District, Dar es Salaam, Tanzania.

**Methods:**

A cross-sectional study was conducted in 20 HIV/AIDS care and treatment clinics. Interviews were conducted with the person in charge and a member of the pharmacy staff from each clinic using a pre-tested semi-structured interview guide. Verbal responses were transcribed, coded and analysed by thematic approach. Quantitative data were analysed using Excel spreadsheet (Microsoft Excel®, Microsoft Corporation).

**Results:**

The total number of clients enrolled in the visited clinics was 32,147, of whom 20,831 (64.8%) had already been initiated onto antiretroviral therapies (ART). Stock-out of antiretroviral drugs was reported in 16 out of the 20 clinics, causing 210 patients to change their ART regimens, during the 12 months preceding the survey. Inefficient supply systems, quantification problems and short expiry duration were cited as the main causes of stock-outs. The coping strategies utilised to prevent changes in ART regimens were: shortening of the refill period, borrowing and moving patients to other clinics.

**Conclusion:**

Changes in ART regimens due to stock-outs of antiretroviral drugs occurred in a small but significant number of patients. This increases the risk of the emergence of drug-resistant HIV strains. Healthcare workers use various coping strategies to prevent changes in ART regimens but, unfortunately, some of these strategies are likely to increase patient-borne costs, which may discourage them from attending their routine clinics, hence leading to unplanned treatment interruptions.

## Introduction

A total of 34 million people were living with HIV/AIDS worldwide and 8 million were already on antiretroviral therapies (ART) by the end of 2011 [1]. Increased access to antiretroviral drugs has brought hope to millions of people living with HIV/AIDS, especially in sub-Saharan Africa. From the mid-2000s to 2011, the number of HIV/AIDS-related deaths in sub-Saharan Africa has declined by 32% [1]. An intensified scaling-up of treatment programs has caused a substantial reduction in HIV/AIDS-related deaths as well as a significant reduction in transmission rates [1,2]. The global target is to ensure that 15 million people have been placed on treatment by 2015 [1].

Tanzania has a population of about 45 million people [3], of whom 1.6 million were estimated to have been infected with HIV by the end of 2012 [4]. The cumulative number of people who had been enrolled in HIV/AIDS care and treatment programs in the country was 1,135,390 and of these, 663,911 (58.4%) were already receiving ART by the end of December 2012 [4]. Since the late 2000s, the Government of Tanzania has been scaling up the enrolment of patients onto HIV/AIDS care and treatment programs through provider-initiated and home-based testing and counselling programs in order to reach more patients [5,6].

By the end of December 2012, about 1,176 approved health facilities, both public and private, were providing HIV/AIDS care and treatment services in Tanzania [4]. The Medical Store Department (MSD) is a public agency which procures, stores and distributes medicines in the public health sector. In 2005, Tanzania adopted a pull system called the Integrated Logistic System (ILS), through which health facilities order their medical supplies from MSD using a single set of procedures. This system also handles supplies for vertical programs such as vaccines and antiretroviral drugs [7,8]. With the ILS system, hospitals order medicines quarterly directly from MSD while dispensaries and health centres order through the District Medical Officers’ (DMO) offices [7].

HIV/AIDS programs are largely funded by donors; some prefer to procure antiretroviral drugs through the MSD, while others use their own agents but rely on the MSD for storage and distribution to the clinics based on their program plans [9]. The MSD, through its extensive distribution network consisting of nine zonal warehouses located in different regions, delivers the ordered medicines directly to the facilities or to the DMO’s office [10]. Thus the performance of ART programs in Tanzania largely depends on the smooth functioning of the MSD. Unfortunately, it faces a number of challenges, some of which are associated with the expansion of the ART programs [11,12].

### HIV/AIDS treatment guidelines in Tanzania

In February 2009, the Ministry of Health and Social Welfare (MoHSW) launched the third edition of its national HIV/AIDS treatment guidelines, which recommended ART to be initiated for all patients with CD4 cell counts less than 200/mm^3^. The default first-line regimen was Zidovudine + Lamivudine + Efavirenz or Nevirapine. Other regimens include: Tenofovir + Emtricitabine + Efavirenz or Nevirapine and Tenofovir + Lamivudine + Efavirenz or Nevirapine [13]. In April 2012, the fourth edition of the guidelines was launched [14], following the WHO’s recommendations of September 2009 which called for the initiation of ART for all patients with CD4 cell counts less than 350/mm^3 ^[15]. This cut-off point has increased the number of patients who are eligible for ART, hence adding more pressure to the already weak and fragile pharmaceutical system.

### Changes in ART regimens due to stock-outs

Changes in ART regimens due to stock-outs of antiretroviral drugs are common, particularly in low-income countries [16,17]. The WHO’s progress report of 2010 reported that stock-outs of antiretroviral drugs were documented in 36 out of the 94 reporting countries [18]. In 2011, Global Fund reported risks of antiretroviral drug stock-outs in 20 African countries, and in one country a number of these drugs were already out of stock [19]. A study by Somi et al. in 2009 in Tanzania reported that 247 patients changed their ART regimens between 2004 and 2007 due to stock-outs of antiretroviral drugs [17]. The National AIDS Control Programme also reported that about 10% of the 7,622 ART regimen changes recorded in 2009 were due to stock-outs [20]. This study aims to explore stock-outs of antiretroviral drugs and further determine the coping strategies employed to prevent ART regimen changes in HIV/AIDS clinics located in Kinondoni district, in Tanzania.

## Methods

### Study type

This was a cross-sectional study conducted in 20 HIV/AIDS care and treatment clinics located in Kinondoni district in Dar es Salaam region. There were 12 public facilities in the sample, seven were private and one belonged to a parastatal institution. Half were hospitals and the rest were primary healthcare facilities. Five clinics were being used as refill centres for patients who had been initiated onto ART elsewhere.

### Study setting

The study was conducted in Kinondoni District, in Dar es Salaam, Tanzania. Dar es Salaam consists of three districts and is the main business city in Tanzania; it also hosts the headquarters of the Medical Store Department. Kinondoni is a typical urban area (531 km^2^) with 27 wards which are further subdivided into 127 hamlets. The district hosts about 1.7 million of the 4.5 million people living in Dar es Salaam [3]. Generally, Dar es Salaam has a poor public transport system consisting of small buses locally called *daladala* with a capacity ranging from 16–35 passengers. During peak hours, usually in the morning and evening, these buses are overcrowded and roads are usually congested.

The prevalence of HIV in Dar es Salaam among people aged 15–49 years in 2011–2012 was 6.9% (9.2% in females and 5.3% in males). As of 2008, the municipality hosted 18 approved HIV/AIDS care and treatment clinics [20] and the certification of more facilities was an ongoing process. At the time when this study was conducted there were about 20 approved clinics in Kinondoni. By December 2010, an estimated 123,345 people living with HIV/AIDS in Dar es Salaam were enrolled in 65 care and treatment clinics and among them 72,513 (58.8%) had already been initiated onto ART [20].

### Data collection and data collection tools

Data was collected from April to May 2011, so this data set is almost three years old; hence the situation may not be the same if a similar study was to be conducted today. Data was mainly collected through in-depth interviews using a pre-tested semi-structured interview guide. Interviews were conducted in the study clinics and recorded by a digital audio recording device.

### Interview guide

The interview guide was composed of two sections; section one for the in charge of the clinic and section two for pharmacy staff (see Additional file 1).

### Study participants

We selected two study participants from each clinic; the person in charge of the clinic and one member of the pharmacy staff. In the hospitals, the heads were mainly medical doctors and the pharmacies were staffed by pharmacists and pharmaceutical technicians. In the dispensaries and health centres, heads were mostly Assistant Medical Officers and frequently nurses were the ones dispensing antiretroviral drugs.

### Collection of quantitative data

Each clinic has a number of tools to record patient information for program monitoring. A pre-treatment register records clients who are visiting the clinics for the first time, whether they are eligible for treatment or not, and the treatment register records clients who have been initiated onto treatment. They also have monthly and quarterly reporting tools which provide information on the cumulative number of patients in care and undergoing treatment.

During our pre-visit, facility managers were given a check-list of questions asking about the number of clients enrolled at their clinics and those who had already been initiated onto ART. In addition, they were asked to estimate the number of patients who had had their ART regimens changed as a consequence of stock-outs during the 12 months immediately preceding our survey.

### Data analysis

A thematic content analysis approach was employed to analyse the qualitative data [21] and the analysis was done manually. Verbal responses from the interviewees were transcribed shortly after each interview. The transcripts were read and re-read several times in order to become familiarised with their content. Transcripts were then coded and similar or related codes were categorised under the same thematic area. Quantitative data were entered into Excel spreadsheets (Microsoft Excel®, Microsoft Corporation) and the outcome variables were reported as absolute numbers and percentages.

### Data validity and ethical issues

To ensure data validity, the importance of the study was emphasised to the informants and they were assured of confidentiality. Phone numbers of the informants were taken so that they could be contacted for clarification or repeat interviews. Ethical clearance was granted by the Ethics Review Committee of the Muhimbili University of Health and Allied Sciences. Permission to collect data in the clinics was sought from the facility managers, and all the informants provided consent to participate in the study.

### Definition of variables

Shortage is defined as a fall in the stock level of antiretroviral drugs below the level needed to maintain patients on their monthly refill period. Stock-out is defined as the complete absence of one or more of the antiretroviral drugs that constitute the ART regimens of the HIV/AIDS patients within the period of 12 months immediately preceding the survey.

## Results

Table 1 gives summary information about the clients enrolled in the clinics. It shows that 32,147 clients were enrolled in the visited clinics, of which 20,831 (64.8%) had already been initiated onto ART. Children aged between 1–14 years accounted for 5.4% of those enrolled and among them, 71.5% had already been initiated onto ART. More females, 22,334 (69.5%), than males, 9,813 (30.5%), were enrolled in the clinics. The female-to-male ratio of those who had already been initiated onto ART was about 2:1.

**Table 1:** Number of clients enrolled in HIV/AIDS care and treatment programs

	**Number enrolled**	**Initiated onto treatment**
Males	9,813 (30.5%)	7,422 (75.6%)
Females	22,334 (69.5%)	13,409 (60%)
Adults (15 years and above)	30,404 (94.6%)	19,585 (64.4%)
Children (1–14 years)	1,743 (5.4%)	1,246 (71.5%)
Total	32,147	20,831 (64.8%)

### Stock-out of antiretroviral drugs and the causes

Stock-outs of antiretroviral drugs were reported in 16 out of the 20 clinics. Of these 16 clinics, 10 reported ART regimen change affecting 210 patients, in five clinics there were no records of those who had changed ART regimens and in one clinic there was no change in ART regimens at all. Regarding the stock-outs, one participant said the following:

*Yes at times antiretroviral drugs run out of stock; for example Efavirenz was out of stock at some point this year and this was a national problem. Therefore those patients who were coming to refill their prescriptions with Efavirenz were given Nevirapine. Some were not happy with this but we had no alternative to keep them on treatments.* (Participant from Facility 12)

The majority of participants mentioned various components of the pharmaceutical supply management system, such as quantification problems, inefficient distribution and expiration due to short shelf-lives as the main reasons for the stock-outs of antiretroviral drugs in their clinics. They said that many problems originate from the supplier side, as elaborated by one participant, who said the following:

*Most of the time I would say the problems begin at the Medical Store Department, because at times you place the order and you are told that certain medicines are out of stock. On other occasions they deliver antiretroviral drugs with very short shelf-lives which expire on the shelves shortly after they have arrived at the facility.* (Participant from Facility 11)

Regarding quantification, the majority of participants said that it is not easy to accurately predict the consumption rate of the medicines as the number of patients initiated onto ART is constantly changing. One participant said:

*When you want to place the order you need to know exactly how many patients will be initiated on antiretroviral treatments in that period, but if the number of patients keeps changing from time to time it becomes difficult to estimate. And if you order too many doses they may expire since some have a short shelf-life.* (Participant from facility 9)

### Coping strategies used to prevent changes in treatment regimens

Healthcare workers in the clinics which experienced shortages and stock-outs of antiretroviral drugs said that they use various strategies, described below, in attempts to prevent the need for patients to change their ART regimens.

#### *Shortening the refill period*

The majority of participants said that when they experience shortages of antiretroviral drugs, the first strategy they use is to shorten the refill period. Thus they give patients a smaller number of pills, usually sufficient to keep them on treatment for two weeks rather than a month. Then, after they have replenished their stock, they give them out on a monthly basis. One participant said the following:

*The major approach we use here when our stock falls is to shorten the refill period; instead of giving them a monthly dose we give them enough for a shorter time so that other patients can also get the medicines. For example, now the refill period for Efavirenz is two weeks instead of one month. If we do it like this every patient will get some pills for some time and if the drugs arrive on time they will receive the usual monthly dose when they come again to refill their prescriptions.* (Participant from Facility 7)

#### *Borrowing from other facilities*

We learned through the interviews that some clinics place larger orders for antiretroviral drugs than they can use in order to cope with irregular increases in patient numbers or stock-outs at the central level. Therefore, it was not surprising that participants, mostly from clinics with a relatively small number of patients, said that they borrow from other clinics which may have a larger stock of the required antiretroviral drugs. One participant said:

*We usually borrow from other nearby centres when we are faced with shortages. It is a normal thing to borrow; once we receive our supplies we return the amount we borrowed. We work by helping each other.* (Participant from Facility 20)

#### *Moving patients to other facilities*

This strategy, although not very common, was also mentioned, especially for those clinics that were established as refill centres in order to minimise congestion at the higher-level facilities. One participant said:

*This clinic is just a refill centre, patients just come here to refill their prescriptions but all the clinical issues are conducted at the hospital. So at times when they come to refill and we do not have the drugs we tell them to go to the hospital where they usually go for their routine clinical check-up.* (Participant from Facility 2)

### What happens when the missing drugs become available again?

All the participants said that usually they inform patients about the reasons for changing their ART regimens and usually they understand and accept it. They said the change can be permanent or temporary depending on the patient’s experience with the new therapy or the availability of the drugs. One participant said:

*The new drugs can cause them side-effects they had never experienced before, so when they come to refill, they complain and ask if the drugs they were using previously have arrived. In this kind of a situation we return them to their previous regimens as soon as we can.* (Participant from Facility 12)

They said, however, that some patients feel more comfortable with their new regimens; hence switching them back becomes impossible.

## Discussion

Shortages of antiretroviral drugs leading to changes in ART regimens were common in the surveyed HIV/AIDS care and treatment clinics. The study also reveals that different coping strategies were used to avoid changes in ART regimens, which is important to reduce the risk of the emergence of drug-resistant HIV strains [22]. Drug resistance increases the risk of rapid disease progression and is of major public health concern [23]. Also, resistance to the first-line antiretroviral drugs imposes large opportunity costs on the treatment programs since second-line antiretroviral drugs are more expensive [24].

Shortening of the refill period was a common strategy utilised to cope with the shortages of antiretroviral drugs. This is important to ensure that patients are maintained on their ART regimens; however, this strategy is associated with increased direct and indirect costs borne by patients and their families in accessing treatment [25]. Both direct and indirect costs in accessing care are associated with poor adherence to treatments [26-28] and treatment interruptions [29]. Treatment interruptions are associated with high rates of drug resistance [30], rapid disease progression and greater numbers of deaths [16,31].

An inefficient supply system and forecasting challenges were cited as the main factors causing shortages and stock-outs of antiretroviral drugs in the clinics. These findings correlate with a Global Fund report which singled out weak forecasting, accelerated patient enrollment and lengthy procurement processes as the main factors behind antiretroviral drug stock-outs in African countries [19]. Lack of adequate skills among healthcare workers to use drugs efficiently, to quantify needs and to manage stocks of antiretroviral drugs have also been reported by several studies in Tanzania [32,33]. It has been argued that the international targets that were set for ART programs greatly over-estimated the capacity of the healthcare systems in sub-Saharan African countries [34].

Recently, the parliamentary committee found large quantities of antiretroviral drugs that had expired at MSD warehouses before being distributed to healthcare facilities, highlighting some of the challenges reported in the facilities. Reliable forecasting, coupled with appropriate inventory management to ensure an uninterrupted supply of antiretroviral drugs, are crucial in the performance of HIV/AIDS care and treatment programs [35].

## Limitations of the study

This study provides a snap-shot evaluation of the performance of the pharmaceutical system, which is under increased pressure due to the scale-up of ART programs in Tanzania. The study employed a purposive sampling method, which limits the generalisation value of its findings since the selected clinics may not give a true representation of all the other HIV/AIDS care and treatment clinics in the country.

The data presented in this paper was based on self-reporting by the participating healthcare workers in the selected clinics. We regret that the methodology used in this paper was not sufficient to collect quantitative data for higher-level analysis. Also, we did not interview patients or officials from the MSD, although we strongly believe they possess important information related to the findings of this study. Their views could have strengthened the main conclusion of this study.

## Conclusions

Changes in ART regimens due to stock-outs of antiretroviral drugs occurred in a small but significant number of patients. This increases the risk of the emergence of drug-resistant HIV strains. Healthcare workers use various coping strategies to prevent changes in ART regimens but, unfortunately, some of these strategies also increase patient-borne costs. This may discourage them from attending their routine clinics, causing unplanned treatment interruptions. Strengthening the supply chain management system is important, otherwise there is a high risk of jeopardising the benefits of antiretroviral treatment programs that have been witnessed in recent years.

## Supplementary Material

Additional file 1 The interview guide. Click here for file
