# Supplementary material for: Stock-outs of antiretroviral drugs and coping strategies used to prevent changes in treatment regimens in Kinondoni District, Tanzania: a cross-sectional study
Source: J Pharm Policy Pract. 2014 Apr 22;7:3. doi: 10.1186/2052-3211-7-3 (PMC4366935; doi:10.1186/2052-3211-7-3)
Supplement: Additional file 1 — The interview guide. [file 2052-3211-7-3-S1.docx]

**The interview guide**

Name of facility:..................

Type of facility:....................

Ownership: A: Public/Government B: Private C: Faith-based D: Other:....................

*Part A: Interview with facility manager*

1. How many people living with HIV/AIDS are enrolled at your clinic and how many are already initiated onto antiretroviral treatments? Segregate them by sex and age.
2. Have you experienced stock-outs of antiretroviral drugs during the last 12 months?
3. How many HIV/AIDS patients have changed treatment regimens due to stock-outs during the last 12 months?

*Part 2: Interview with a member of pharmacy staff*

1. Have you experienced stock-outs of antiretroviral drugs during the past 12 months?
2. If yes, what was the cause of the stock-out?
3. What coping strategies do you use to prevent changes in treatment regimens when you face shortages or stock-outs of antiretroviral drugs?
4. Do you inform patients of the reasons for changing their treatment regimens? If so, how do they react to that information?
5. For those patients who change regimens, what do you do when their previous antiretroviral drugs become available again at your clinic?
